# Supplementary material for: The impact of R&D factors flow and regional absorptive capacity on China’s economic growth: Theory and evidence
Source: PLoS One. 2024 Nov 11;19(11):e0310476. doi: 10.1371/journal.pone.0310476 (PMC11554203; doi:10.1371/journal.pone.0310476)
Supplement: Attachment — Submitted filename: 01-Response to Reviewers.docx [file pone.0310476.s002.docx]

**Response to Reviewer 1 Comments**

Reviewer #1: It is my pleasure to review the manuscript for the esteemed journal. The manuscript studies the relationship between R&D factors flow and economic growth and explores the moderating effect of regional absorptive capacity on the relationship between R&D factors flow and economic growth from theoretical and empirical perspectives. The topic of the paper is important and worthy of investigation. Undeniably, the study could have contributed significantly if it had been developed, deepened, and broadened. However, the manuscript needs further improvement before it is accepted for publication. The reviewer has listed some specific comments that might be helpful for the author to enhance the quality of the manuscript further. Please consider the particular comments listed below.

Letter to Reviewer #1

Dear Reviewer #1,

We sincerely thank you for your thorough review of our manuscript. We found them very useful to improve the quality of the manuscript. We fully appreciate your precious time and effort. Please accept our sincerest gratitude. We have responded to all your comments, and all revisions have been highlighted in the updated manuscript.

Warm regards,

Corresponding Author

Comment 1: Abstract. The abstract is well-written. However, it should further underscore the scientific value added to your paper in your abstract.

Response 1: Thank you for your recognition of this paper. Your insightful comments have been invaluable in enhancing this paper. Therefore, we have added the scientific value in the abstract. The details are as follows.

“

This article reveals that the positive impact of the inflow of R&D factors on spatial spillovers and economic growth varies depending on regional absorptive capacity.

”

**See rows 47 to 48, or page 3 of the manuscript.**

Thank you again for your valuable guidance and feedback.

**Kind regards.**

Comment 2: Sections of Introduction. The novelty of this paper should be further justified by highlighting the main contributions to the existing Introduction and Literature review. The application of emerging technologies can be seen as a typical R&D innovation. Please consider citing the following paper: entitled “Influence of artificial intelligence applications on total factor productivity of enterprises—evidence from textual analysis of annual reports of Chinese-listed companies.”; next entitled “Relationship between financial development and intelligent transformation of manufacturing: evidence from 69 countries.”, next entitled “The impact of AI on carbon emissions: evidence from 66 countries.”, and next titled “Impact of economic policy uncertainty shocks on China's financial conditions.”. There has already been a large number of literatures related to the research on the impact of innovation on the economy and society. Therefore, it should be better to elaborate on the contribution of the work to the existing literature.

Response 2: We sincerely thank you for your valuable comments. Our primary focus lies in examining the dynamic flow of R&D factors among regions from the perspective of factor flows, which differs from the perspective of the investment or accumulation of R&D factors. Therefore, after carefully reading the literature you provided, I have added them to the Introduction section of this manuscript. To clarify that innovation is very important and worth investigating. The details are as follows.

“

As an inexhaustible driver of economic growth, innovation can facilitate sustainable development by enhancing total factor productivity, accelerating the optimization and upgrading of the economic structure, and reducing energy consumption and carbon emissions [1-3].

”

**See rows 62 to 65 of the manuscript.**

I have added the fourth article to the section on prospects for future research in the article. (See the last paragraph of the manuscript.)

Next, existing literature on the relationship between R&D factors flow, spatial knowledge spillovers, and economic growth is limited, and we have identified only four relevant papers: Fujita and Thisse (2003), Bai et al. (2017), Audretsch and Belitski (2020), and Wang et al. (2021). Note that Wang et al. (2021) is a new addition to this revised manuscript.

“

Wang, et al. [8] used the data of China from 2008 to 2018 and empirically found that R&D personnel and R&D capital inflows have a significant positive impact on regional economic growth, but the inflow of R&D personnel into the region has a significant negative spatial spillover effect on surrounding areas [8].

”

**See rows 107 to 111 or page 6 in the manuscript.**

This addition serves to further highlight that the existing literature on the relationship between R&D factors flow, spatial knowledge spillovers, and economic growth has not considered a crucial variable: regional absorptive capacity.

The significance of studying R&D factors flow lies in the fact that the flow of R&D factors generates spatial knowledge spillovers, contributing additional value to the economy. However, regional absorptive capacity, as an influential variable affecting knowledge spillovers, undoubtedly impacts the process through which R&D factor flow affects economic growth via spatial knowledge spillovers. But existing literature has overlooked it.

Neglecting regional absorptive capacity implies that the same amount of R&D factor flow would result in the same knowledge spillovers and economic growth for all regions. However, in reality, different regions have varying capabilities to absorb external knowledge. Therefore, the inflow of R&D factors into different regions yields diverse outcomes in terms of knowledge spillovers and economic growth. Consequently, incorporating regional absorptive capacity into the model is reasonable and is worth exploring. We provide further elaboration on this matter in the Introduction section of the manuscript. The details are:

“

However, existing research on the impact of R&D factors flow on spatial knowledge

spillover and regional economic growth has overlooked the role of regional absorptive

capacity. This implies that the knowledge spillover and economic growth resulting from the flow of the same amount of R&D factors are assumed to be uniform across all regions, which is not the case, thus providing an opportunity for this study to investigate.

”

**See rows 111 to 115, or the last sentence of the third paragraph of the Introduction section, or page 6 in the manuscript**.

Next, in order to demonstrate that existing literature on the relationship between knowledge spillovers, absorptive capacity, and economic growth has not explored the perspective of R&D factor flow, we have summarized the key points of Borensztein (1996) and Jung and López‐Bazo (2017) in the relevant section. Because these two articles are representative. Moreover, we added Kaneva and Untura (2019). This addition serves to further emphasize that the existing literature on the relationship between knowledge spillovers, absorptive capacity, and economic growth has not taken into consideration the aspect of R&D factor flow. The details are as follows.

“

Borensztein, et al. [11] argued that the actual impact of knowledge spillovers on economic growth depends not only on the volume of spillovers but also on the absorptive capacity of the economic entities[11]. Jung and López‐Bazo [12] demonstrated that the process of knowledge spillover promoting economic growth relies on regional absorptive capacity[12]. Kaneva and Untura [13] suggested that the lagging regions fail to experience growth from knowledge spillovers due to their limited absorptive capacity[13].

”

**See rows 121 to 127, or page 7 in the manuscript**.

Furthermore, we have added a corresponding statement in the concluding part of the fourth paragraph in the Introduction, emphasizing that existing literature on the relationship between knowledge spillovers, absorptive capacity, and economic growth has not considered the aspect of R&D factor flow. The details are as follows.

“

From this, it can be seen that research on the correlation between regional absorptive capacity, knowledge spillover, and economic growth is relatively abundant. However, these literatures have overlooked the fact that the flow of R&D factors is the cause of spatial knowledge spillover.

”

**See rows 143 to 146, or the last sentence of the fourth paragraph of the Introduction section, or page 8 in the manuscript**.

Please note that R&D factor flow and spatial knowledge spillovers are not the same; the former is one of the significant factors contributing to the latter.

Finally, in the fifth paragraph of the Introduction section, we have added two summarizing statements as follows.

“

In summary, the existing literature has separately investigated the impact of R&D factors flow on spatial knowledge spillover and economic growth or the influence of regional absorptive capacity on spatial knowledge spillover and economic growth. However, there is a limited amount of research literature that comprehensively analyzes the interplay among these four variables within a unified framework.

”

**See rows 147 to 151, or the first two sentences of the fifth paragraph of the Introduction section in the manuscript**.

Consequently, this study combines the two types of research mentioned above into a unified framework.

What’s more, regarding the main contributions of this paper, you can refer to our response to your Comment 3.

Your expert guidance has indeed greatly enriched our paper, and we sincerely appreciate your advice.

**Best regards!**

Comment 3: The introduction section needs to give more prominence and clarity to the primary contributions, and it is recommended that the authors elaborate on this section and consider adding some additional contributions.

Response 3: We sincerely thank you for your valuable comments. Therefore, in the sixth paragraph of the introduction, we have reorganized and further elaborated on the innovative points of this article, and added an innovative point. The detailed content is as follows.

“

The main innovations of this article can be conveyed in three points.

First, we introduce regional absorptive capacity into the augmented knowledge creation and diffusion model of Bai, et al. [6]. We analyze the relationship between regional absorptive capacity and R&D factors flow, knowledge spillovers, and economic growth. Our findings indicate that varying regional absorptive capacities result in diverse knowledge spillovers and economic growth when the same quantity of R&D factors flow in, thereby aligning the knowledge creation and diffusion model more closely with reality.

Second, we improve the methodology for measuring regional absorptive capacity, expanding Zahra and George’s [17] four-dimensional measurement of absorptive capacity into a comprehensive index system consisting of 31 indicators. This enhancement ensures a more scientific and accurate assessment of regional absorptive capacity.

Third, we consider the directionality of R&D factors flow, and from the perspective of R&D factors inflow, we employ spatial panel econometric models to analyze the spatial spillover effects of regional absorptive capacity and R&D factors inflow on economic growth, further taking into account the moderating role of regional absorptive capacity in this relationship.

”

**See rows 161 to 175, or page 9 in the manuscript.**

**Kind regards.**

Comment 4: In the sections on Calculation and characteristic analysis of China’s provincial regional absorptive capacity, I would suggest that the authors show some of the elements of the principal component analysis measure in detail. For example, how many principal components were extracted? What is the cumulative contribution? This may be more convenient for readers to understand.

Response 4: Undoubtedly, only reviewers well-versed in Principal Component Analysis could offer such professionally insightful suggestions. We greatly appreciate your input on this matter. Consequently, we have revised the relevant section of our paper about introducing Principal Component Analysis. The detailed content is as follows.

“

Our PCA result received four principal components, with eigenvalues of 17.72, 4.74, 2.07, and 1.34, respectively. Their cumulative contribution rate is 0.83, and their uniqueness is less than 0.6. Therefore, they meet the selection criteria for principal components.

”

**See rows 357 to 360, or page 19, or the Calculation and characteristic analysis of China’s provincial regional absorptive capacity section of the manuscript for details.**

**Warm regards.**

Comment 5: In the Robustness test section, it is unknown whether the authors can add ways such as changing the measurement of the dependent variable and the core explanatory variables to verify whether the same conclusion as the above empirical results can be obtained. Of course, this is a suggestion; I wonder if it's feasible for the author's reference.

Response 5: Without a doubt, only reviewers with a strong background in econometrics could provide such professional recommendations. Replacing core variables is one of the important robustness testing methods.

As regional absorptive capacity is our core variable, we followed Cohen and Levinthal’s (1990) approach and conducted a robustness check by using R&D intensity to represent absorptive capacity. The results confirm the robustness of our findings.

**See rows Column (5) in Table 9, or pages 39 to 40 in the manuscript.**

Note that, here, we no longer present the results without considering regional absorptive capacity because it is the same as Model (1) in Table 7.

We are very lucky to be able to obtain this result by effort.

**Yours sincerely.**

Comment 6: The Conclusion and Policy Implications section should be pushed more towards discussing rather than summarizing the results. An in-depth discussion of the study's limitations and prospects for future research is welcome. Also, an indication of theoretical and practical implications would add significant value.

Response 6: We sincerely appreciate your valuable feedback.

We have revised the previous general policy recommendations because they were not sufficiently detailed (See rows 691 to 712, or page 42 in the manuscript).

In order to enhance the academic and practical value of our paper, we have conducted more discussions on the conclusions (See rows 713 to 734, or page 43 in the manuscript).

We have provided a detailed introduction to the shortcomings of this article and potential future research directions (See the last paragraph of the manuscript).

**Best wishes!!!**

**Response to Reviewer 2 Comments**

Reviewer #2: This paper investigates an interesting phenomenon about the impact of R&D factors flow and regional absorptive capacity on China’s economic Growth. The manuscript has been written well in large part. However, some revisions are required in this work.

Letter to Reviewer #2

Dear Reviewer #2,

We sincerely thank you for your thorough review of our manuscript. We found them very useful to improve the quality of the manuscript. We fully appreciate your precious time and effort. Please accept our sincerest gratitude. We have responded to all your comments, and all revisions have been highlighted in the updated manuscript.

Warm regards,

Corresponding Author

1. The innovation of the article should be added in the abstract.

Response 1: We sincerely thank you for your valuable suggestion. We agree to add the innovation points of our article in the abstract. The details are as follows.

“

The novelty of this article is to introduce regional absorptive capacity into the theoretical model, refine the methodology for assessing regional absorptive capacity in empirical research, and examine its moderating effect between the inflow of R&D factors and regional economic growth.

”

**See rows 43 to 47 in the manuscript.**

Your valuable suggestions have greatly helped to improve the quality of this article, and we are very grateful.

**Kind regards.**

2. Abstract should include a brief statement about the policy implication.

Response 2: We sincerely thank you for your valuable comments. We have modified the content of the policy statement in the abstract to:

“

The study provides valuable policy insights for accelerating the flow of innovation factors, enhancing regional absorptive capacity, and consequently promoting long-term sustainable economic development in the region.

”

**See rows 48 to 51 of the manuscript for details.**

**Warm regards.**

3. I didn't see any innovation in the paper. The author should clearly indicate the innovation expenditure of the paper.

Response 3: We sincerely thank you for your valuable comments. Therefore, in the sixth paragraph of the introduction, we have reorganized and further elaborated on the innovative points of this article, and added an innovative point. The detailed content is as follows.

“

The main innovations of this article can be conveyed in three points.

First, we introduce regional absorptive capacity into the augmented knowledge creation and diffusion model of Bai, et al. [6]. We analyze the relationship between regional absorptive capacity and R&D factors flow, knowledge spillovers, and economic growth. Our findings indicate that varying regional absorptive capacities result in diverse knowledge spillovers and economic growth when the same quantity of R&D factors flow in, thereby aligning the knowledge creation and diffusion model more closely with reality.

Second, we improve the methodology for measuring regional absorptive capacity, expanding Zahra and George’s [16] four-dimensional measurement of absorptive capacity into a comprehensive index system consisting of 31 indicators. This enhancement ensures a more scientific and accurate assessment of regional absorptive capacity.

Third, we consider the directionality of R&D factors flow, and from the perspective of R&D factors inflow, we employ spatial panel econometric models to analyze the spatial spillover effects of regional absorptive capacity and R&D factors inflow on economic growth, further taking into account the moderating role of regional absorptive capacity in this relationship.

”

**See rows 161 to 175, or pages 8 to 9 in the manuscript.**

**Kind regards.**

4. What is the connotation of R&D factors flow and regional absorptive capacity？

Response 4: Thank you very much for your questions about the definition of R&D factors flow and regional absorptive capacity. I am happy to explain them.

R&D factors refer to the talent, funds, technology, and resources used for innovation and the mechanisms formed by their combination. R&D factors flow refers to the flow of R&D factors, such as talent migration between regions and the transfer of innovation funding between regions.

We added the explanation of R&D factors flow in the manuscript. The details are as follows.

“

The flow of R&D factors mainly includes two key components: interregional talent migration and the transfer of innovation funding.

”

**See rows 79 to 80, or page 5 in the manuscript**.

Next, regional absorptive capacity refers to a region's knowledge absorptive capacity. Knowledge absorptive capacity initially referred to a firm’s ability to acquire, digest, transform, and apply external knowledge. Scholars later extended it to the regional and national levels.

We added the explanation of regional absorptive capacity in the manuscript. The details are as follows.

“

Subsequently, researchers extended this concept to regional levels, defining regional absorptive capacity as the integrated ability of a region to acquire, digest, transform, and apply external knowledge[10].

”

**See rows 117 to 119,or page 6, or the second sentence of the fourth paragraph of the Introduction section in the manuscript**.

**Warm regards.**

5. Since the analysis of the absorption capacity characteristics of provincial-level regions in China is involved, it is recommended to add spatial evolution analysis, which can be referred to in the following literature.

Yaping Xiao, Dalai Ma , Fengtai Zhang, et al. Spatiotemporal differentiation of carbon emission efficiency and influencing factors: From the perspective of 136 countries.[J].Science of the Total Environment.,2023,879:163032.

Response 5: We sincerely thank you for your valuable recommendation and the suggested article. We have incorporated spatial evolution analysis into our research, following the example provided in the referenced literature. The inclusion of map visualizations has enhanced the clarity and intuitiveness of our data presentation, making it more accessible for readers to understand the absorption capacity characteristics of provincial-level regions in China. We appreciate your guidance in this regard. However, per the journal’s requirements, the images are uploaded separately, and I'm unsure whether you can view them directly. Please feel free to reach out to us at any time.

**See page 23, or the paragraph before the Emperial methods section, or the supporting figure files.**

**Best wishes!**

6. The data sources are too general, and different variables' data sources should be explained.

Response 6: We appreciate your observation regarding the data sources description. You are right, and we acknowledge the importance of providing more specific and detailed information about the sources of different variables. In response to your feedback, we have revised the manuscript to include explicit explanations of the data sources, ensuring greater transparency and clarity in our presentation. The details are as follows.

“

The telephone penetration rates data were sourced from the Ministry of Industry and Information Technology website (https://www.miit.gov.cn/gxsj). The R&D personnel and R&D capital data were sourced from the China Science and Technology Statistics Yearbook. The geographical distance data were sourced from China’s National Platform for Common Geospatial Information Services (https://www.tianditu.gov.cn). The marketization comprehensive index data were sourced from the China Market Index Database (https://cmi.ssap.com.cn). The remainder of the data were sourced from the National Bureau of Statistics of China (http://www.stats.gov.cn). The handling method for missing data is as follows: (1) The telephone penetration rates for the years 2008 and 2009 are computed by summing the fixed-line telephone penetration rate and mobile phone penetration rate for the fourth quarter of each respective year. (2) For the year 2010, six indicators including full-time equivalent R&D personnel in industrial enterprises above designated size, internal R&D expenditures industrial enterprises above designated size, the number of R&D projects, the number of new product project item, funding for developing new products, and sales revenue of new products are represented as the average values of the corresponding indicators for the years 2009 and 2011.

”

**See page 29, or the Data sources section in the manuscript.**

**Warm regard.**

7. Why does the Moran Index value appear to be the same for multiple years? The reason should be explained.

Response 7: We appreciate your insightful question regarding the consistency of the Moran Index values across multiple years. Actually, they are different.

The reason why Moran I appeared the same in the previous manuscript is that we only displayed numerical values up to three decimal places. Taking your valuable suggestion into consideration, in this version of the manuscript, we have displayed the values of Table 4 to four decimal places, which clearly demonstrates their differences.

**See page 30 or Table 4 in the manuscript.**

**Kind regards.**

8. Findings of the paper should be related to previous studies. albeit, some effort has been made in this regard, it is highly selective.

Response 8: We appreciate your feedback on the relevance of our findings to previous studies.

Our study builds upon the foundation laid by Bai et al. (2017). However, they neglected the role of regional absorptive capacity. Thus, we innovate by incorporating regional absorptive capacity into the framework of R&D factor flow, spatial knowledge spillovers, and economic growth. The theoretical analysis and empirical research in this paper contribute by integrating regional absorptive capacity, R&D factor flow, spatial knowledge spillovers, and economic growth within the same framework.

The inclusion of regional absorptive capacity in the model is of significant importance. Assuming the exclusion of regional absorptive capacity implies that, for all regions, the same quantity of R&D factors inflow would result in identical spatial knowledge spillovers and economic growth. However, in reality, different regions possess varying absorptive capacities, leading to different conversion capabilities for external knowledge. Consequently, the spatial knowledge spillovers and economic growth resulting from the inflow of R&D factors into different regions will vary.

After considering your valuable feedback, we have compared our findings to the existing literature. The details are as follows.

“

Next, we will engage in a discussion based on our conclusions.

First of all, the overall findings of this study regarding the impact of the flow of R&D factors on economic growth through full-sample regression are generally consistent with Bai, et al. [6], suggesting that the flow of R&D factors has a promotional effect on the economic growth of the local region as well as neighboring regions. However, the analysis of regional heterogeneity in this study indicates that, in terms of the direct growth effects, the inflow of R&D factors into the eastern region leads to the greatest economic growth, followed by the western region, while the central region experiences the smallest gain. This pattern aligns closely with the respective absorptive capacities of these three regions. In other words, the impact of the flow of R&D factors is more significant in areas with higher absorptive capacity. In comparison, in areas with weaker absorptive capacity, the effect of the flow of R&D factors is less pronounced.

In the second place, regarding the conclusions regarding spatial spillover effects, our study differs from Caragliu and Nijkamp’s [10] research. Caragliu and Nijkamp’s [10] study suggests that the greater the absorptive capacity of the local region, the smaller the knowledge spillover to the surrounding areas. In contrast, our research indicates that the greater the absorptive capacity of the local region, the larger the spatial spillover effects on the surrounding areas. This difference may be attributed to the choice of models in our study, which differs from the model chosen in Caragliu and Nijkamp’s [10] research. Caragliu and Nijkamp’s [10] study used specific indicators to measure outward knowledge spillovers and treated them as explained variables in the SAR model. In contrast, our spatial knowledge spillover was derived from the spatial effects decomposition of the SDM model, which was selected based on tests like LR and Wald.

”

**See page 43, or the penultimate paragraph of the manuscript.**

**Warm regard.**

9. The policy recommendations are too general, and the feasibility and operability of the policy recommendations should be improved.

Response 9: We appreciate your valuable feedback on the policy recommendations in our document. Your observation is indeed very insightful. We acknowledge that the policy recommendations in our previous manuscript version were somewhat general.

To address this issue, we will strive to improve the feasibility and operability of these suggestions, making them more practical and effective. We have made serious revisions to the policy recommendations section.

“

The findings of this study offer profound policy recommendations for implementing the strategy of innovation-driven development and achieving long-term sustainable economic growth. On the one hand, the interregional R&D factors flow should be encouraged, and this can be achieved by focusing on the following three aspects. First, relax the restrictions on the settlement of talent, such as lowering the settlement threshold, increasing registered residence quotas, and expanding eligibility criteria to attract highly skilled individuals to migrate. Second, reducing the settlement cost of talent, for instance, by increasing the relocation subsidies for incoming talent to address the housing problem of introduced skilled workers. Third, strengthen exchanges and cooperation between regional R&D institutions. For example, the government should incentivize researchers from different areas to co-apply for national and provincial grants, including open research topics within nationally designated laboratories. On the other hand, the ability of regions to absorb, transform, and utilize external knowledge should be enhanced, especially in lagging areas. This can be accomplished from three perspectives. Firstly, increase investment in education, improve the quality of education, and enhance human capital development, such as establishing high-level universities, strengthening the construction of teaching staff, and improving the education and teaching system. Secondly, the government should offer enterprises more favorable tax policies for scientific research and provide more R&D funding support. This helps the region consolidate its ability to absorb foreign knowledge while promoting technological innovation. Thirdly, transportation and information infrastructure should be improved, and interregional population, knowledge, and trade connections should be strengthened. This will reduce information asymmetry while creating more opportunities for knowledge inflow.

”

**See rows 691 to 712, or page 42 in the manuscript.**

10. English expression needs further polishing.

Response 10: Thank you for your valuable feedback and suggestions for improving the English language in the manuscript. We have once again polished the English expression in this paper. In fact, as non-native English-speaking researchers, conveying our intended meaning accurately requires continuous effort and revisions. We are eager for these revisions to meet the standard of language expression.

Thank you very much!

**Kind regards.**

11. The article should further point out the shortcomings and limitations.

Response 11: We appreciate your suggestion regarding the limitation of our article. We have incorporated this aspect into our manuscript. The details are as follows.

“

Lastly, our study has limitations in measuring R&D factors flow. For the mobility of R&D personnel, this study only selected wage levels as the primary influencing factor. However, it could also be influenced by other factors, such as environmental pollution levels[29]. Similarly, for the flow of R&D capital, this study only considered the average profit rate as the primary influencing factor. However, in reality, it could also be affected by the magnitude of investment risk[30]. While these limitations may affect the size of the estimated coefficients in the regression analysis, they do not change the conclusion that the inflow of R&D factors can positively impact the economic growth of the local region and its neighboring areas. Thus, future research could incorporate additional influencing factors that attract the flow of R&D factors and provide a more precise measurement of R&D factors flow.

”

**See the last paragraph in the manuscript.**

Your feedback has been valuable in enhancing the quality of our work.

**Yours sincerely.**

**Best wishes!**
