# Supplementary material for: The impact of R&D factors flow and regional absorptive capacity on China’s economic growth: Theory and evidence
Source: PLoS One. 2024 Nov 11;19(11):e0310476. doi: 10.1371/journal.pone.0310476 (PMC11554203; doi:10.1371/journal.pone.0310476)
Supplement: Attachment — Submitted filename: 01-Response to Reviewers.docx [file pone.0310476.s003.docx]

**Response to Reviewer 3 Comments**

**Letter to Review 3**

Dear Review 3,

We sincerely thank you for your thorough review of our manuscript. We found them very useful to improve the quality of the manuscript. We fully appreciate your precious time and effort. Please accept our sincerest gratitude. We have responded to all your comments, and all revisions have been highlighted in the updated manuscript.

Warm regards,

Corresponding Author

===========================================================================

Comment 1: Improve the abstract by adding an attractive background at the start of the paper. Also, include the main objective briefly, include period of investigation, and the sample of the study. Also, present the main findings followed by a sentence about policies.

Response 1: Thank you for your suggestions. We have added them in our abstract. The details are as follows:

(1) We have added the background at the start of the paper, which reads:

“

Innovation is the source of economic growth. Innovation in a region comes from its own knowledge creation and knowledge spillovers from other regions. Previous studies showed that R&D factors flow benefits knowledge spillover, thereby promoting economic growth. But these studies ignored the impact of a region’s knowledge-absorptive capacity on knowledge spillovers. Ignoring the impact of regional absorptive capacity means that the knowledge spillover from the same R&D factors flow is the same, clearly inconsistent with reality.

”

(2) We have added period of investigation, and the sample of the study in our abstract, which reads:

”

Second, … , utilizing panel data of 30 provinces in China from 2008 to 2021.

”

(3) We have presented the main findings followed by a sentence about policies, which reads:

“

According to the conclusions above, enhancing regional absorptive capacity is equally important as facilitating the flow of R&D factors. Therefore, it is vital for a region to strengthen its absorptive capacity for new knowledge while promoting R&D factors flow.

”

**Kind regards.**

Comment 2: Explain the advantages of the research methodology used in the context of this study. The appropriateness of the research methodology for this work should be provided.

Response 2: Thanks. **We added them on page 24, section 4.1.3 of the manuscript, which reads**:

“

Bai, et al. [9] shows that the flow of R&D factors among provinces is not independent of each other, and the flow of R&D factors to a certain province may be affected by the economic behavior of other provinces, and ignoring the spatial correlation accompanied by the flow of R&D factors may lead to the wrong setting of the model [9]. In addition, Ertur and Koch [22] show that there is also a spatial spillover effect on the knowledge absorptive capacity between regions[22]. Based on that, …

”

**Kind regards.**

Comment 3: The data section should be improved by following some international papers. All data sources must be referred in the text as well as in the reference list.

Response 3: Thanks. We checked the data section again and made some modifications, including listing them in the reference.

**Please see page 19, section 3.2; page 28, section 4.2.2.; page 48, references 25,26,27,32,33.**

**Kind regards.**

Comment 4: The contribution should be provided in a separate paragraph at the end of the introduction. Besides, this section should have a detailed background, the significance of the research, the justification of the sample, and the policy-related significance of the study.

In this section, conduct a critical evaluation of past literature, aiming to identify a literature gap that justifies the necessity of this study. This evaluation should be thorough, examining recent studies published within the last few years to ensure relevance and comprehensiveness. Below are some suggested studies to expand this section:

https://doi.org/10.1016/j.jclepro.2020.125529 0959-6526

https://doi.org/10.1007/s11356-021-17673-2

Response 4: Thanks. This suggestion is very helpful in improving the introduction section of our article. According to your suggestions, we improved the introduction section of our manuscript.

First of all, we have provided our contributions at the end of the introduction. **The details can be seen on page 9 of the manuscript.**

It is worth noting that we have expanded our contributions based on the comment#5 of the additional editor.

Secondly, we have expanded the introduction section part of our study. The specific modifications we have made are as follows:

**We have added a reference on page 5, which reads:**

“Some scholars argued that R&D factors are essential to secure economic interests and develop innovative technologies[4-6].

”

Below are the literatures that we added:

doi: 10.1016/j.techfore.2019.04.007.

doi: 10.1007/s11356-021-17673-2.

doi: 10.1371/journal.pone.0299697.

These literatures are relevant to emphasize the necessity of the R&D factors.

**We have added a reference on page 7, which reads:**

“

Ahmed, et al. [17] stated that absorptive capacity plays an important role in spreading knowledge and economic growth[17].

”

Below is the literature that we added:

doi: 10.1016/j.jclepro.2020.125529.

The addition of this reference helps to emphasize that studying the effect of regional knowledge absorption capability on knowledge spillover is of great importance.

**Kind regards.**

Comment 5: Improve the policy directions of the study.

Response 5: Thanks. We have improved the policy directions of our study. The details are as follow:

“

The policy implications are as follows. (1) The interregional R&D factors flow should be encouraged, thereby unleashing both the direct and spillover effects of such flows to bolster economic growth. In terms of stimulating the R&D personnel flow, the government should relax the restrictions on the settlement of talent, such as lowering the settlement threshold, increasing registered residence quotas, and expanding eligibility criteria to attract highly skilled individuals to migrate. Additionally, the government should reduce the settlement cost of talent, for instance, by increasing the supply of talent apartments and enhancing the relocation subsidies for incoming talent to address the housing problem of introduced skilled workers. In terms of fostering the R&D capital flow, the government should strengthen exchanges and cooperation in innovative activities among regions. For example, efforts should be made to incentivize cross-regional applications for open topics in national key laboratories and joint applications by researchers from different regions for various levels and types of projects, including national and provincial natural science funds and social science funds. (2) Enhancing the ability of regions to absorb, transform, and utilize external knowledge, the moderating effect of regional absorptive capacity can be unleashed. This can be accomplished from three perspectives. Firstly, since education and human capital are important influencing factors of absorptive capacity, we can improve regional absorptive capacity by increasing investment in education, improving the quality of education, and enhancing human capital development, such as establishing high-level universities, strengthening the construction of teaching staff, and improving the education and teaching system. Secondly, the government should offer more favorable tax policies for scientific research and provide more R&D funding support for enterprises, and encourage their R&D investment. According to the dual role of enterprise R&D, this helps to improve the absorptive capacity of the enterprise, which in turn helps to strengthen the basic ability of the region to absorb foreign knowledge. Thirdly, efforts should be made to further improve transportation and information infrastructure, enhance interregional population, knowledge, and trade connections, creating more opportunities for knowledge inflow, thereby enhancing the region’s ability to identify and absorb knowledge.

”

**Please see pages 42 to 44 for details.**

We sincerely thank you again for your valuable comments, which have been very helpful in improving our article.

**Besh wishes!**

**Wishing you happiness every day!**

**Response to Additional Editor**

===========================================================================

**Letter to Additional Editor**

Dear Additional Editor,

We sincerely thank you for your thorough review of our manuscript. We found them very useful to improve the quality of the manuscript. We fully appreciate your precious time and effort. Please accept our sincerest gratitude. We have responded to all your comments, and all revisions have been highlighted in the updated manuscript.

Warm regards,

Corresponding Author

===========================================================================

Comment 1. Please update the relevant literature review.

Response 1: We have diligently conducted another thorough review of the recent literature about our research topic, ensuring both the relevance and comprehensiveness of the references cited in our manuscript. The specific modifications we have made are as follows:

**We have added a reference on page 5, which reads:**

“Some scholars argued that R&D factors are essential to secure economic interests and develop innovative technologies[4-6].”.

These literature are relevant to emphasize the necessity of R&D factors.

**We have added a reference on page 7, which reads:**

“Ahmed, et al. [17] stated that absorptive capacity plays an important role in spreading knowledge and economic growth[17].”.

The addition of this reference helps to emphasize that it is important to study the regional knowledge absorption capability.

**Kind regards.**

Comment 2. Please add theoretical and policy level implications.

Response 2: We sincerely thank you for this valuable comment. We have added the theoretical implications of this study. Moreover, we have improved the policy implications of this study.

First of all, for theoretical level implications, we have added that:

“

The theoretical implication is that the knowledge brought by the flow of R&D factors would not automatically lead to spillovers. In fact, varying regional absorptive capacities result in diverse knowledge spillovers and economic growth when the same quantity of R&D factors flows in.

”

**Please see it on page 42, the last sentence of section 6 of the manuscript.**

Secondly, we have improved our policy implications:

“

The policy implications are as follows. (1) The interregional R&D factors flow should be encouraged, thereby unleashing both the direct and spillover effects of such flows to bolster economic growth. In terms of stimulating the R&D personnel flow, the government should relax the restrictions on the settlement of talent, such as lowering the settlement threshold, increasing registered residence quotas, and expanding eligibility criteria to attract highly skilled individuals to migrate. Additionally, the government should reduce the settlement cost of talent, for instance, by increasing the supply of talent apartments and enhancing the relocation subsidies for incoming talent to address the housing problem of introduced skilled workers. In terms of fostering the R&D capital flow, the government should strengthen exchanges and cooperation in innovative activities among regions. For example, efforts should be made to incentivize cross-regional applications for open topics in national key laboratories and joint applications by researchers from different regions for various levels and types of projects, including national and provincial natural science funds and social science funds. (2) Enhancing the ability of regions to absorb, transform, and utilize external knowledge, the moderating effect of regional absorptive capacity can be unleashed. This can be accomplished from three perspectives. Firstly, since education and human capital are important influencing factors of absorptive capacity, we can improve regional absorptive capacity by increasing investment in education, improving the quality of education, and enhancing human capital development, such as establishing high-level universities, strengthening the construction of teaching staff, and improving the education and teaching system. Secondly, the government should offer more favorable tax policies for scientific research and provide more R&D funding support for enterprises, and encourage their R&D investment. According to the dual role of enterprise R&D, this helps to improve the absorptive capacity of the enterprise, which in turn helps to strengthen the basic ability of the region to absorb foreign knowledge. Thirdly, efforts should be made to further improve transportation and information infrastructure, enhance interregional population, knowledge, and trade connections, creating more opportunities for knowledge inflow, thereby enhancing the region’s ability to identify and absorb knowledge.

”

**Please see it on pages 42 to 44 of the manuscript.**

Thank you again for your valuable guidance and feedback.

**Kind regards.**

Comment 3. Manuscript need editing, please ensure to edit the manuscript.

Response 3: We have made corresponding edits to the article to ensure its logic and fluency.

**Warm regards.**

Comment 4. The conclusion should be extended, and contribution should be justified.

Response 4: We have rechecked and improved the conclusion section of this article, and the specific modifications are as follows:

**We have added a sentence on page 41, which reads:**

“

In 2008, 2015, and 2021, the absorptive capacity of the Eastern region measured 1.75, 3.55, and 5.75, respectively. Over the same years, the absorptive capacity of the Central region were 0.68, 1.53, and 2.43, respectively and those of the western region were 0.51, 1.55, and 2.59, respectively. The significant difference in regional absorptive capacity in China suggests that the effect of unit R&D factor inflows on regional economic growth will vary depending on regional absorptive capacity.

”

**We have added a sentence on page 42, which reads:**

“

The direct and indirect effects of R&D personnel inflow accounted for 12.121% and 87.878% of the total effect, respectively. Meanwhile, the direct and indirect effects of R&D capital inflow accounted for 9.302% and 89.583% of the total effect, respectively.

”

This sentence extends the previous sentence.

**We have added a sentence on page 42, which reads:**

“

Fourth, from the spatial perspective, the moderating effect of regional absorptive capacity is significant.

”

We believe that this helps to serve as the starting sentence of our conclusion#4.

**Moreover, we have added a sentence in the penultimate sentence of Section 6, which reads**:

“

According to the conclusions above, we find that enhancing regional absorptive capacity is equally important as facilitating the flow of R&D factors.

”

**Kind regards.**

Comment 5. Contribution need to be extended.

Response 5: This comment is valuable. We have extended the contribution section of this article, and the details are as follows:

We change the starting sentence of this paragraph, which reads:

“

Our study is distinguished from other studies and makes contributions to the literature in the following aspects.

”

**Please see it on page 9, the last paragraph of this section.**

**In addition, we have extended the contribution on page 9, which reads:**

“

In the study of Bai, et al. [9], authors have regarded that the knowledge brought by the flow of R&D factors can automatically lead to spillovers. They have not considered the impact of a region's knowledge absorptive capacity on knowledge spillover effects. This means that the spillover effects brought by the inflow of unit factors are the same for all regions, but in fact, they are different.

”

We sincerely thank you again for your valuable comments, which have been very helpful in improving our article.

**Besh wishes!**

**Wishing you happiness every day!**
