# Supplementary material for: The impact of R&D factors flow and regional absorptive capacity on China’s economic growth: Theory and evidence
Source: PLoS One. 2024 Nov 11;19(11):e0310476. doi: 10.1371/journal.pone.0310476 (PMC11554203; doi:10.1371/journal.pone.0310476)
Supplement: Attachment — Submitted filename: 01-Response to Reviewers.docx [file pone.0310476.s004.docx]

**Response to the editorial office**

**Letter to the editorial office**

Dear the editorial office,

We sincerely thank you for your thorough review of our manuscript. We have noted your concerns about similarity and fully understand them. Based on your suggestion, we have included an overview of Wan et al.'s article in the introduction section of our paper and conducted some discussion. Our article differs significantly from Wan et al.'s article. Below, we will elaborate on the differences between our work and the Wan et al. article you mentioned.

===========================================================================

Response to the editorial office:

Although both Wan et al and our team study R&D factor flow (also referred to as R&D elements flow), and both of us use R&D factor flow as the independent variable in our empirical tests, our articles differ significantly and focus on different aspects. The details are as follows:

(1) In terms of research content, Wan et al. study the impact of R&D factor flow on the innovation capabilities of China's hi-tech industries, as well as the moderating role of government intervention between R&D factor flow and the innovation capabilities of China's hi-tech industries. In contrast, our research focuses on the impact of R&D factor flow on regional economic growth in China, and the moderating role of regional knowledge absorptive capacity between R&D factor flow and China's economic growth.

(2) In terms of research subjects, Wan et al. use the innovation capabilities of China's hi-tech industries as the dependent variable, whereas we use China's economic growth as the dependent variable. Wan's study focuses on the industry level, while our research is conducted at the regional level. We believe our study is broader and more macro in scope.

(3) In terms of research focus, Wan et al emphasize government intervention and use it as the moderating variable in their study, whereas we focus on regional knowledge absorptive capacity and use it as the moderating variable in our paper. One of the innovations of our research is that existing literature on R&D factor flow and economic growth does not consider the impact of regional knowledge absorptive capacity. Our study addresses this gap. We propose and demonstrate that the contribution of unit innovation factor inflow to regional economic growth varies depending on the regional knowledge absorptive capacity.

(4) In terms of research methods, in the theoretical analysis section, Wan et al use narrative descriptions to explain the impact of R&D factor flow and government intervention on the innovation capabilities of China's hi-tech industries. However, we not only use narrative descriptions but also employ mathematical models to provide mathematical proof, illustrating the impact of R&D factor flow and regional knowledge absorptive capacity on China's regional economic growth.

(5) In terms of research methods, in the empirical study, Wan et al. simply used the proportion of government funds to R&D funds as a proxy variable for government intervention. However, we constructed a comprehensive index system to measure regional knowledge absorptive capacity. We presented the measurement results of the knowledge absorptive capacity of different provinces in China, which helps readers analyze and study the knowledge absorptive capacity across various Chinese provinces.

(6) In terms of research methods, in the empirical study, Wan et al. employed the SAR model from spatial econometrics for the analysis. In contrast, we utilized the SDM model from spatial econometrics for our analysis. Renowned spatial economist Elhorst has noted that the advantage of the SDM model is its ability to simultaneously capture both spatial lag and spatial error effects, providing a more accurate description of the relationships in spatial data. The SDM model can capture the spatial spillover effects of the independent variable (R&D factor flow) on the dependent variable, which the SAR model cannot. The importance of studying the spatial spillover effects of R&D factor flow lies in the fact that R&D factor flow benefits not only the economic growth of the recipient region but also the economic growth of neighboring regions, which the SAR model cannot demonstrate.

(7) In addition, Wan et al.'s study did not conduct a heterogeneity analysis, whereas we also performed a heterogeneity analysis. Therefore, our content is more comprehensive.

(8) Finally, it is important to point out the issue of keyword translation. We chose to translate it as "R&D factor flow" rather than "R&D elements flow" because we believe that "factor" is more aligned with the common economic terminology for production factors in English. Of course, as native speakers, you may have a more accurate judgment on this matter, and I am open to your suggestions.

Based on your concerns and valuable suggestions, we have added a brief overview of Wan et al.'s article to the introduction section of our manuscript:

“

Wan, et al. [12] argued that both R&D personnel flow and R&D capital flow have significantly promoted China's hi-tech industry innovation capabilities, while government intervention exerts a negative regulatory impact between R&D factor flow and hi-tech industry innovation capabilities [12].

”

See lines 120 to 123 in the introduction section of our manuscript.

To facilitate a concise comparison, we have added the following sentences to the contributions section of the introduction in our manuscript:

“

Fourth, different from Wan et al.’s [12] study, which focuses on the impact of R&D factors flow and government intervention on innovation capabilities and employs the spatial autoregressive (SAR) model to demonstrate this [12], our research uses the SDM model to examine the relationships between R&D factors flow, regional absorptive capacity, and China's economic growth. Methodologically, the SDM model allows for the decomposition of the direct effects and spatial spillover effects of the independent variable (R&D factors flow), leading to more accurate spatial estimates compared to the SAR model.

”

See lines 195 to 201 in the introduction section of our manuscript.

Regarding the references, we have updated Wan et al.'s article from [35] in the old manuscript to [12] in the new manuscript, resulting in corresponding adjustments to the other reference numbers.

**We assure you that our manuscript is original and innovative. If you have any further questions, please feel free to reach out to us, and we will be happy to address them.**

Warm regards,

Corresponding Author

===========================================================================

Appendix:

Key Differences

|  | Wan et al’s work | Our work |
| --- | --- | --- |
| Research content | The impact of R&D factor flow on the innovation capabilities of China's hi-tech industries, as well as the moderating role of government intervention between R&D factor flow and the innovation capabilities of China's hi-tech industries. | The impact of R&D factor flow on regional economic growth in China, and the moderating role of regional knowledge absorptive capacity between R&D factor flow and China's economic growth. |
| Independent variable | R&D factor flow | R&D factor flow |
| Dependent variable | Hi-tech industries’ innovation capacity | Regional economic growth |
| Moderating variables  (Research focus) | Government intervention | Regional absorptive capacity |
| Empirical research method | SAR model, captures only the direct effects of the independent variable. | SDM model, captures both the direct effects and the spatial spillover effects of the independent variable. |
